# Hospital and Institutional News

**Published:** 1917-02-03

**Authors:** 


### HOSPITAL AND INSTITUTIONAL NEWS.

### MORE GIGANTIC LEGACIES FOR HOSPITALS.

MR. WILLIAM HERRING, who died on December 8, 1916, aged 89, brother of the late Mr. George Herring, has left estate valued at £840,362. He has left about £56,000 direct to charities, besides an enormous residue which will ultimately be similarly distributed, in all probability. Dr. Barnardo's Homes receive the largest specific bequest—£25,000. The London Hospital comes a good second with £10,000; in addition to which Lord Knutsford is left a private legacy of £2,000. The Haven of Rest, Maidenhead, is left £5,000, and the Metropolitan Hospital Sunday Fund £1,000. The residuary estate, amounting to about three-quarters of a million (£750,000) is left to the trustees "with the request and in the confidence that they will dispose of the same in accordance with any letter, memorandum, or paper deposited with the will." It is not yet disclosed whether any such memorandum exists, or what charities it indicates; but it is stated that this expression of the testator's wishes "is not to derogate from the absolute and unconditional bequest to the trustees, or to impose on them any trust, obligation, or responsibility." If the said trustees do not want to be swamped by applications from energetic hospital secretaries and boards of management, they had better publish without delay any memorandum of Mr. Herring's wishes upon which they propose to act. On the same day as Mr. Herring's benefactions were published, there appeared also particulars of the will of Mr. Otto Baerlein, who has left about £30,000 for such charitable purposes at Alderley Edge as the Urban District Council there may direct. Alderley Edge is a comparatively small community, if recollection serves us; possibly, if local needs do not require the whole £30,000 (which it is scarcely likely they can), the Urban Council might be induced to start a hospital for soldiers with any balance there may be.

### LORD RHONDDA AND QUACK PRACTICE.

ON January 24 the President of the Local Government Board (Lord Rhondda, formerly Mr. D. A. Thomas) received a deputation from a number of local authorities and associations in reference to the treatment of venereal disease by unqualified practitioners. Some of the medical representatives present took advantage of the occasion to mention that in their opinion quack practice of all kinds should be suppressed. In replying to the various members of the deputation who had put forward their views, Lord Rhondda naturally confined himself mainly to the subject of unqualified practice in venereal disease, and dropped a fairly broad hint to the effect that he hopes to hinder or suppress it. Later, when replying to a vote of thanks, he alluded to the possibility that self-interest might be charged against doctors when advocating the prohibition of quack practice. He said such charges would not influence him in the slightest degree, and that he was perfectly satisfied that the medical representatives were actuated by considerations of State policy and not of self-interest. On the same day Lord Rhondda also received a deputation from the Women's Freedom League, which is anxious that the treatment of women suffering from venereal diseases should be in the hands of women doctors.

### THE FRENCH SYSTEM OF CARE FOR THE DISABLED WOUNDED.

AT the request of the present Prime Minister, when Minister of Munitions, Sir Henry Norman has visited France in the capacity of "liaison officer" in order to study the French system of care and after-care of the disabled in war. His report has now been published, and is worthy of study by those interested in this aspect of the treatment of war injuries. Sir Henry is inclined to be critical of some parts of the French system, especially of the lack of centralisation of authority and organisation, upon the need for which he is very emphatic. In order to secure proper co-ordination, and to avoid overlapping, he suggests a central committee under a Minister. On this committee there should be representatives of the War Office, one or more Labour and Trade-Union representatives, an educationist with a special knowledge of technical training, an employer of manual labour, two women with special experience of the care of soldiers, representatives of benevolent societies (*e.g.*, the Soldiers and Sailors Help Society), and at least two scientific medical men who are specialists in the treatment of these cases. This committee, Sir Henry Norman suggests, should control all schools and institutions and all money. Training should begin while the patient is in hospital. The French system of re-education for previous or kindred employment is favoured as the most economical.

### THE RENEWAL AND MAINTENANCE OF ARTIFICIAL LIMBS.

IT was announced recently in the press that the Order of St. John of Jerusalem (in England) has undertaken by request of the War Office and the Admiralty to be responsible for the repair and renewal of artificial appliances for wounded sailors and soldiers. In amplification of that announcement, the Secretary-General of the Order (Mr. Evelyn Cecil) has been authorised by the Grand Prior (the Duke of Connaught) to explain the special objects which the Order has in view. These are (1) to maintain and repair artificial limbs and surgical appliances; (2) to render this assistance as far as possible at or near to the men's homes, and while they continue in their appointment; and (3) to ensure that full use is made of the appliances provided at Government expense, and that no man remains on crutches or otherwise unfit for employment when an artificial limb or appliance would enable him to become a useful member of the community. In support of these eminently practical and business-like aims, it is contemplated by the Order in due course to make a public appeal. In relieving the War Office and the Admiralty of this work the Order is both carrying out its own best traditions and deserving well of the community: the whole arrangement is much to be commended from every possible point of view, including that of the mutilated themselves.

### THE NATIONAL RELIEF FUND AND HOSPITALS.

The fourth report of the Executive Committee of the National Relief Fund to the Prince of Wales on the administration of the Fund for the six months ending September 30, 1916, states that the total contributions received to that date amounted to £5,634,385. Add to this the interest on sums temporarily invested, and amounts collected for the Queen's "Work for Women" Fund, the aggregate receipts at the date mentioned may be stated in round figures at six million pounds. On the other side of the account nearly three millions have been spent on naval and military relief and about half a million on civil relief. It is stated that so far as the industrial classes are concerned there is practically no distress, except in the case of watering-places on the East Coast. Nothing has been given to ordinary hospitals; in the early days of the war the Executive Committee came to the decision that these institutions were not eligible for support from the Fund, but since then so many applications have been received from the hospitals that it was thought advisable to reconsider the matter. However, the second review of the situation left the committee unrelenting and the hospitals still out in the cold.

### HOW WILL THEY SPEND THE MONEY?

The work of supplementing separation allowances payable by Government must have cost the Relief Fund a large amount during the first two years of the war, but as these allowances have now been increased, to correspond with the higher cost of living, the Fund will be relieved of an important and expensive part of its work. If it should happily prove that the Zeppelins and errant warships will soon have enough of East Coast reception, the East Coast should quickly recuperate. Thus the Fund will have two of the larger pensioners off its hands. What is to be done with the £2,500,000 in hand? Perhaps the committee are wishful to be prepared for the unknown conditions that will immediately follow the war, for although they are pledged to spend all the funds in the relief of distress caused by this war and not to hoard up for any other purpose, it is not expected of them that they should have empty coffers the day after peace is declared. But with all these possible requirements in mind, a quarter of a million judiciously distributed this year amongst those voluntary hospitals that have been worst hit would not hurt the Fund, and would, we are sure, be quite within the measures of relief that the subscribers to the Fund intended to support.

### WAR CHARITIES AND CHURCH FINANCE.

The correspondence in our columns on the subject of church collections, and the deductions occasionally made from them for church expenses, is timely and should lead to a reconsideration of principle and practice in the matter. The objections to the practice of deductions need no further emphasis, but The Hospital has always been conscious of the difficulties of the churches in this matter. Hospital Sunday originated the custom of devoting one Sunday collection to national charity, and its success has led the organisers of later movements to claim other Sunday collections for other purposes. Hence in part the difficulty which faces those responsible for church collections. We contend, however, that the energy which the voluntary hospitals have had to expend in maintaining their income from voluntary gifts should set the churches an example which they have been slow to follow. Church finance seems uncongenial to most clergy, and the average churchwarden appears to use little of his business experience as a layman to help out the financial problems of the church of which he is the voluntary guardian. He presents a balance-sheet, does the routine work honestly and quietly, occasionally issues an appeal; but that is all. We would respectfully suggest, therefore, that clergy, and especially churchwardens, should remember that voluntary hospital finance was once conducted on these generally simple lines, but that they had to be much extended: by studying the arts of appeal; the organisation of weekly collections; the way to create and maintain subsidiary organisations, and the like. In face of the claims made on the churches these methods can no longer be neglected; and it is neglect of them on which criticism of want of energy is bound to fasten.

### A LARGE-MINDED COLLIERY COMPANY.

A generous offer on the part of the Ebbw Vale Colliery Company to provide and partly maintain a cottage hospital for the Abertillery district was announced last week. One condition of the gift is that the institution shall cost between £25,000 and £30,000, and that it shall be so equipped with appliances and instruments for the alleviation of suffering as will place it in the very front rank. The company have left the whole of the arrangements, including the drawing up of plans, etc., in the hands of a committee of twenty-five members, of whom nine are to be their representatives, and the remainder direct representatives of the workmen. When the hospital is erected the company undertake to find half of the cost of its maintenance. The institution is to be open to the tradespeople and residents of the district who contribute towards its upkeep, equally with the company's workmen. A joint committee has been sitting for over a year now charged with the task of securing a cottage hospital for Abertillery, and this solution of the problem is accepted as most gratifying.

### AN OPPORTUNE REORGANISATION SCHEME.

Following the resignation last week of the post of secretary of the King Edward VII. Welsh National Memorial Association by Mr. Gwilym Hughes—who has accepted a post in London in connection with Mr. Neville Chamberlain's

National Service scheme—a readjustment is to be made in the duties of the existing staff in order to obviate the necessity of appointing an official in the place of Mr. Hughes. The proposal made by the Finance Committee is that Mr. D. W. Evans shall combine the duties of director and legal adviser; that Mr. F. J. Alban, recently appointed controller, shall become secretary as well; and that Mr. Edwin Radford, the assistant secretary, shall be the deputy controller and deputy secretary. A reorganisation of the administrative staff of the Association has been "in the wind" for some time, and the recommended changes are not likely to be vetoed by the Council of the Association.

### FRICTION AT BEVERLEY.

A meeting of the subscribers of the Beverley Dispensary and Hospital is being called to consider the remarkable situation which has arisen at the institution. In August last all the members of the hospital's medical staff—with the exception of Dr. Munro—sent in their resignations. Dr. Munro remained, discharging single-handed and without remuneration the duties of medical officer. These facts, among others, were placed before the governors at the annual meeting last week. One governor wanted to know what caused the resignation of the medical staff, and Mr. Geoffrey Warburton, chairman of the directors, replied that four of the staff resigned because they refused to associate with a certain gentleman. The state of affairs, he added, was most unfortunate, and the directors would welcome any suggestion of a way out of the difficulty—short, of course, of placing any reflection upon Dr. Munro. The meeting thought the best suggestion it could offer was the remission of the problem to the subscribers.

### THE SUPPRESSION OF DECEPTIVE ADVERTISEMENTS.

In the same issue of the *Times* in which the views of a deputation to Lord Rhondda were reported, advocating the suppression of advertisements of quack remedies and practitioners in connection with venereal diseases, a prominent advertisement on the front sheet of that paper gave proof that it is not only the unqualified who disseminate deceptive and dangerous information. The title of the announcement in question, the only "displayed" and large-type advertisement on the whole sheet, was "The Futility of Operations in Cancer." It purported to emanate from "A Consulting Physician," whose book gives "proofs that dietics [*sic*] and hygienics provide the only potent remedy." From the fact that this advertisement has not been repeated during the few days that have since elapsed, it may possibly be that the editor of the *Times* has taken advice and has refused any longer to insert it. We sincerely trust that this is the case; but if it is not so, and if he is in any doubt as to the propriety of "Consulting Physician's" action, it would be well for him to take the opinion of the able and energetic writer on medical subjects whose articles in the *Times* have lately aroused so much interest (as well as opposition) in medical circles. The medical profession looks forward with hope and confidence to the day when medical or other treatment will supersede operations in the relief and cure of cancer. But that day has not come yet, and it is, in our opinion, wholly contrary to public policy that the public should be influenced to believe that it has. The zeal of advertisement managers has placed newspaper editors in false positions before to-day, and we should be happy to believe that that is what happened in this case, and that the editor of our leading daily paper had no knowledge of the use his columns were being put to.

### TWO IMPORTANT POINTS AS TO COMPULSORY SEGREGATION.

Reporting to the Southwark Borough Council, the Public Health Committee says it has for some time past had under consideration questions raised in committee (*a*) whether a sanitary authority has power, in any circumstances, compulsorily to remove from the home an infectious case of tuberculosis; (*b*) whether under Sections 12 and 20 of the Children Act of 1908 the sanitary authority has power to remove children living in contact with an infectious parent or person suffering from tuberculosis. The Medical Officer of Health and the Tuberculosis Officer, to whom the matter was referred, state that the Public Health (London) Act of 1891, Section 66, gives power to the local authority to remove to hospital a person suffering from an infectious disease who is without proper lodging and accommodation. The local authority, however, has seldom put this section into force, as it is very difficult to convince a magistrate that a patient in an ordinary home is without proper lodging and accommodation, and if it is difficult in ordinary cases of infectious disease, how much more difficult would it be in a case of tuberculosis. With regard to question 2, Section 12 of the Children Act states that if any person over the age of sixteen years so exposes a child or young person in a manner likely to cause such child or young person unnecessary suffering or injury to his health that person shall be guilty of a misdemeanour and liable to a penalty. The officers are of opinion that it would be straining this section very much to apply it to contacts, and it could only be applied in such cases as that of a consumptive parent or person who, after being advised that spitting about the room would endanger the health of a child or young person, neglected to take the necessary precautions.

### MUNICIPAL HOSPITALS.

In a letter to the Urban District Councils Association regarding the admission of cases to isolation hospitals, the Local Government Board write pointing out that under Section 131 of the Public Health Act, 1875, the sanitary authorities have a general power to provide hospitals for the sick inhabitants of their districts. The power is not limited to cases of infectious disease. The letter proceeds to explain that, although originally hospitals provided by sanitary authorities were intended chiefly to protect the public against the spread of infection, this view has since been modified. Having regard to the great social convenience and advantage of the accommodation in these hospitals it has gradually been realised that, especially for infectious diseases, the interests of the community and of the individual are identical. But at the same time the Board consider that a ratepayer has not an unconditional right to the removal to hospital of a case of infectious disease occurring in his house, even if the hospital is not full.

### "ECONOMY" OVER HEALTH AT MERTHYR.

ON the ground that the rates for the borough of Merthyr are the highest in the country, there were indignant protests at a meeting of the local Health Committee last week, when a recommendation was brought forward for the appointment of an assistant health inspector. Some of the members declaimed heatedly against the proposed "extravagance," and there was much talk of "soft jobs" being provided for public officials, who ought to be made to work harder. In vain did the chairman point to the regulations of the Local Government Board, and he warned the committee that if they persisted in the suggested economy the inevitable result would be the dissemination of more disease. The desire of the so-called economists apparently was to make the Health Committee a scapegoat for the high rates—a most unfair procedure, for, as Dr. A. Duncan (the medical officer) pointed out, Merthyr's Health Department is one of the cheapest in the district. Eventually the appointment of the health inspector was passed by a majority, but there were angry murmurings that those who voted for it ought to have their names recorded as being responsible for the exorbitant rates.

### THE CONSUMPTION OF WINE IN THE FRENCH ARMY.

SOME astonishment has been aroused in this country by certain official returns as to the quantity of wine supplied to the French Army by the French Government, and consumed by the soldiers. It appears that in 1915 the Government requisitioned and purchased six hundred and eighteen million large bottles of red wine for this purpose, and that this enormous amount was nearly doubled in 1916. There need be little astonishment when it is remembered that the allowance per man is almost a pint a day. These red wines—the *vin ordinaire* of the country—are of low alcoholic content; but all the same, the total represents an enormous quantity of alcohol. The medical authorities of the French Army are satisfied that the allowance helps to keep the troops in condition, and that its advantages greatly outweigh any disadvantages. Probably the greatest advantage of all is that these wines are far less likely than is the water supply in any theatre of war to be polluted by the germs of disease. The drinking of unboiled water on the battlefield, more particularly when trench war is going on, is highly dangerous; and the most elaborate sanitary precautions, including preventive inoculation, are of less avail than the drinking of those fluids alone which are bacteriologically safe. Tea, properly made with none but boiling water, is one such safeguard; but the average Frenchman is not used to tea-drinking as is the average Englishman, and his favourite *vin ordinaire* is a substitute equally efficacious in avoiding the cause of dysentery, enteric, and other water-borne diseases.

### TYPE-READING FOR THE BLIND.

AT the meeting of the Röntgen Society on Tuesday next, Dr. Fournier d'Albe, Physicist to the Board of Inventions and Research, is to demonstrate his type-reading optophone by which blind persons are enabled to read ordinary letterpress by ear. This is done by audible telephone currents, produced by intermittent light of various musical frequencies. The printed sheet is passed over a slab upon which is an aperture where a horizontal beam of light is projected from an illuminated siren disc, and by means of a set of selenium bridges exposed to light reflected from the type, sounds which vary with the shape of each letter are carried into a telephone. Some practice is required before the blind person can read with any speed, but the inventor states that the alphabet can be learned in three or four days, and with practice a speed of twenty-five or more words a minute can be acquired. Dr. Fournier d'Albe also proposes to show other properties and applications of selenium, including its direct action, in which one lamp is made to light another without a relay. The meeting will be held in the Institute of Electrical Engineers on Tuesday next at 8.15 P.M. Visitors interested in the subject will be welcomed on this occasion.

### IS A COLLECTOR WORTH HIS SALT?

A CERTAIN Midland hospital has had some success with a collector. In December 1913, we learn, a collector, in addition to a local chartered accountant, who acts as secretary, was appointed at a salary of £100 a year, out of which he had to provide a cycle and bear the cost of its wear and tear. Since then the income appears to have doubled, owing largely to the collector's success in organising "Days," for which a free hand was given to him in 1915. In 1916 the ladies, who do all actual collecting on the "Days" in question, were allowed to do the organising themselves. Whether the public was tired of "Days" or whether they were amateurs at organisation we do not know, but the proceeds of the "Days" dropped considerably. At a recent monthly meeting of the board of management the application of the collector to have his wages advanced to 50s. was refused, as well as an application for a £10 extra bonus for the past year. Among the reasons given were depreciation of "Days," that the increase of subscriptions was only £300 a year, and, finally, that the board was paying the market value of a collector, at which sum, it was averred, as good or better a collector could be got.

### THE COLLECTOR VERSUS THE TRAINED SECRETARY.

THE answer to the question raised in the preceding paragraph is not that the market value of a collector is so much more or less than £100 a year, but that the practice of having a collector and a part-time secretary is obsolete and should be discontinued. The old plan means that the part-time secretary keeps the minutes, does the accounts and other routine work, while the outside collector raises the money. This is bad, since it tends to make the so-called secretary a routineer with no ideas on hospital policy, and necessarily limits the collector's horizon to the amount of money which he can raise. The arrangement of these functions so divided is out of place in a modern hospital, which has, or should have, no use for routineers and "out-for-profit" seekers. "Days" are all very well; but, like other purely rule-of-thumb methods, they cannot form the basis of hospital finance. For this is needed a local public interested in every branch of its hospital, and actively working for it through many subsidiary organisations. No collector can create these. He can only impose a series of wiles upon the public, for he has no continuous strain of interest and co-operation on which to work. A secretary is the man for this work; a collector and a part-time secretary have given way in all up-to-date hospitals of any size to a trained administrator, to whom finance is a part of hospital policy and not merely the latest art of "raising the wind."

### THE SUNDAY SHAVE IN HOSPITALS.

AN excited discussion took place at a recent meeting of the Dewsbury Board of Guardians on the question whether temporary permission should be given to the barber of the infirmary to do some of his work on Sundays. After various members had protested, saying that the institution should set a better example than that of allowing shaving and haircutting on Sundays, and that the barber would occupy his time more usefully in attending a place of worship, Mr. P. H. Wilson replied that the arrangement, which was due to shortage of labour, would cease after the war. The old saying that the Sabbath was made for man and not man for the Sabbath is too deep for most of us, as the above discussion shows. But to deprive a patient of the barber on Sunday, in war-time, when needs are great and workers few, is not an alternative that recommends itself as exemplary.

### THE SANITARY INSPECTOR'S SALARY.

THE schoolmaster and the sanitary inspector have arrived at the time when the lip service which their professions receive is being accompanied by an exposure of the low salaries often paid to them. An alert correspondent in Sunday's *Observer* was able to contrast the salaries offered to schoolmasters and window-cleaners, to the disadvantage of the former, and quoted the text of advertisements in support of his case. The sanitary inspector is often able to live only by combining a variety of occupations more or less allied to sanitary inspection itself. Now, too, that the emoluments which have sometimes made the net income larger than the nominal salary paid are stated to be rapidly diminishing, demands for an increase in salary are becoming frequent, and we do not think that their approval can be long delayed. When sanitary inspection is given a better status, and is designed to enforce a higher standard, the work of our medical officer of health departments should be strengthened to solve the larger issues for which they properly exist.

### BABY SAVING IN WALES.

IT is a source of satisfaction to know that even comparatively small towns, such as Llandudno, are taking their part in the efforts now being put forth to preserve infant life. Last week, at the annual meetings of both the Cottage Hospital and the Charity Association at the North Wales resort, consideration was given to the question of child welfare, and appropriate practical steps taken. At the latter meeting Dr. E. R. Woodhouse pointed out the great scarcity of qualified midwives, and proposed the appointment of a third nurse, who should be a trained maternity nurse, for the Great Orme district. Letters supporting the proposal were read from every medical practitioner in the town, and it was unanimously adopted by the meeting. Lord Mostyn presided at the Cottage Hospital's yearly gathering, when the board of management was instructed to consider the provision of beds for difficult maternity cases.

### THE MENTALLY DEFECTIVE AT PLYMOUTH.

THERE is apparently some dissatisfaction locally that Plymouth has not set about dealing with the problem of its mental defectives who are over school age in a really practical manner. Alderman Littleton, the chairman of the Education Committee, presented a strong indictment at a conference last week of head-teachers in the borough. He asserted that, although the empowering Act has been in operation two years, in Plymouth virtually nothing has been done. Within the past two or three weeks several aggravated cases of mental deficiency have been brought to the notice of the magistrates, but no useful step can be taken because there is not even an institution to which this type of case can be sent. The provision of a home where those who are a danger not only to themselves but to society is one of Plymouth's most pressing needs, according to the chairman of the Education Committee.

### MILK STERILISATION CRITICISED AT LIVERPOOL.

A MEDICAL member of the Liverpool Corporation made an attempt, at a meeting of the City Council last week, to pull down what he called "the false god of sterilised milk." The estimates of the spending committees for the next financial year were under consideration, and the Health Committee's proposed expenditure of £300,000 occasioned some discussion. Dr. T. W. Byrne entered into a severe criticism of the Corporation's depôts for the supply of sterilised milk for infants, declaring that they were still worshipping the false god of sterilised milk. The policy of the Health Department was stoutly championed by Alderman Shelmerdine, who claimed that sterilised milk has been the means of saving from 20,000 to 30,000 children in Liverpool. This saving of life cost about £2 2s. per baby, and was well worth it. Dr. Byrne did not carry the lay members of the Council with him; for the estimates, as presented, were approved.

### THE "COCAINE AND OPIUM REGULATIONS" AND INSTITUTIONS.

When the amended Order was issued regarding the keeping and supply of cocaine and opium, we drew attention to the anomalous position of the hospital pharmacist who, by the terms of the Order (not being engaged in "*retail*" business), was not, as in the original Order, an "authorised person" under the Act. The Home Office has now, in response to representations from the Pharmaceutical Society and Institutional Pharmacists' representatives, issued a general permit giving qualified pharmacists engaged in dispensing at hospitals permission to act as "authorised persons" by virtue of their qualification. The Order reads: "In pursuance of the Defence of the Realm (Consolidated) Regulations I hereby grant to all persons employed or engaged at any public hospital or other public institution, being persons duly registered under the Pharmacy Act, 1868, a permit to purchase cocaine and opium for the use of the hospital or other institution as aforesaid in accordance with the conditions prescribed by the Regulations." Pharmacists will still require to conform to the stringent regulations requiring the keeping of records of all supplies given out at hospitals, etc. Apparently, unless the dispenser is a *registered pharmacist* he has no authority to act as "authorised," and in that case the medical officer must keep and be responsible for the cocaine and opium (solid) in use at his institution.

### GET GOOD STOCKS OF CLINICAL THERMOMETERS.

The hospital administrator is becoming quite familiar with the address of the Minister of Munitions. Should the present spell of cold weather reveal deficiencies in the heating system and additional radiators be needed, for the supply of labour and material the consent of the Minister is required. Those who have desired to purchase hospital furniture of steel construction will have experienced a correspondence on the question of the substitution of wood or other material for steel in satisfying their requirements. The latest difficulty is in respect of clinical thermometers. There was a time when competition to secure orders from hospitals for these necessary articles was very brisk; nowadays the hospital needing more than the very few to be obtained by retail purchase must go first to the Optical and Glassware Department of the Ministry at 119 Piccadilly, with the order to the manufacturer in duplicate, and if the department is sympathetic the order is endorsed and the duplicate kept for filing purposes. Naturally, the hospital desiring to be put to as little trouble as possible, and wishing to protect itself against unknown shortages in the clinical thermometer market of the future, will take good care to obtain permission to order an abundant supply.

### ANALYSIS OF DRUGS.

The County of London Insurance Committee have decided to add a proviso in their agreement with chemists and druggists supplying drugs and appliances to insured persons that they shall sell to any of the persons mentioned in the Sale of Food and Drugs Act, 1875, any drug or medicine prescribed for the use of insured persons. Some difficulty has arisen through the working of the Act, and it is anticipated that this addition to the agreement will dispose of it. Dr. R. K. Brown, Medical Officer of Health for Bermondsey, is of opinion that the complicated procedure of the Act renders the examination of medical prescriptions extremely difficult, and in many cases impossible. If the sample was divided into three parts the quantities, as a rule, were much too small for quantitative analysis. A far better way would be for the Insurance Committee or the Commissioners to have their own analyst to take samples under agreement with the chemists. Penalties could be provided for the discovery on analysis of any breach of agreement, and, no doubt, under such an agreement special methods would soon be adopted by analytical chemists for the examination of medical prescriptions. The views of the Medical Officer have been forwarded to the Insurance Commissioners and the County of London Insurance Committee for their consideration.

### THIS WEEK'S DRUG MARKET.

On the whole, business in the drug market is quiet, with few price changes of a remarkable character. As was not unexpected, the price of quicksilver has been advanced, but up to the time of writing there had been no alteration in the quotations for calomel, corrosive sublimate, and other salts of mercury; it would not be surprising, however, if makers of these products raised their quotations. In face of the strong demand for honey, prices still tend upwards. Myrrh is scarce and becoming dearer. A good business has been done in gamboge at advanced rates. The scarcity of cocaine has become more pronounced, and prices are again higher. Phenazone is again dearer, in spite of the arrival of small supplies from a neutral country. The downward tendency in the value of phenacetin continues. Nothing has occurred to relieve the scarcity of barbitone, and anything offered fetches very high prices. The high value of camphor is well maintained. Bromides are unchanged.

### TO OUR READERS.

Contributions are specially invited from any of our readers to these columns. They should deal with topical subjects and news. They must be **authenticated for the information of the Editor only. The minimum payment if published is 5s.** There is no hard-and-fast rule as to space, but **notes of about twenty lines in length are preferred.**

